# Open Globe Injuries Related to Traffic Accidents: A Retrospective Study

**DOI:** 10.1155/2021/6629589

**Published:** 2021-02-04

**Authors:** Phit Upaphong, Pongsant Supreeyathitikul, Janejit Choovuthayakorn

**Affiliations:** Department of Ophthalmology, Faculty of Medicine, Chiang Mai University, Chiang Mai, Thailand

## Abstract

**Purpose:**

To evaluate epidemiology, clinical characteristics and outcomes of patients sustained traffic-related open globe injury (OGI).

**Methods:**

The medical records of all OGI patients who were admitted in a tertiary referral center from January 2006 to December 2016 were retrospectively evaluated. Then, the records of injuries related to traffic accidents were identified and reviewed in detail.

**Results:**

Among the overall causes of OGI, traffic-related accidents comprised 92/978 (9%) of cases. Nearly half of the injuries (51%) occurred in the 20–39-year-old group and 59% involved automobile transportation. Globe rupture occurred in 48 (47%) eyes. Following treatments, LogMAR visual acuity (VA) significantly improved from a median (interquartile range) of 2.3 (1.9–2.3) to 1.7 (0.3–3.0), at the final appointment. Presence of relative afferent pupillary defect and presence of retinal detachment were predictors for poor final visual outcomes.

**Conclusions:**

Traffic-related OGI had a high prevalence in the young. The risky transportation modes were motorcycles in teenagers and automobiles in young adults. Despite treatment, there was a considerable proportion of impaired final VA. This information could help establish effective safety education and encourage regular adherence to road safety behaviors in the high-risk groups.

## 1. Introduction

Open globe injury (OGI), defined as a full-thickness laceration of the eye wall, is one of the main conditions leading to an acquired visual disability and a subsequent decline in quality of life [1]. Previous studies from New Zealand and Singapore estimated the annual incidence of OGI to be 2.8 and 3.7 per 100,000 population [2, 3]. There are several etiologies of OGI which have been reported with different characteristics [4–6]. Among these, traffic-related OGI has been consistently described as one of the most common preventable injuries [7–11]. Within the overall cases of OGI, about 2 to 17.6% were related to traffic accidents. [12–16].

As there are limited data, this study aimed to assess the demographics, ophthalmic clinical characteristics, and outcomes of severe open globe injuries related to traffic accidents. The findings may contribute useful data in establishing appropriate healthcare education for traffic-related OGI.

## 2. Patients and Methods

This retrospective study was performed at Chiang Mai University Hospital, a tertiary referral center in Northern Thailand, and conducted in accordance with the Declaration of Helsinki. The protocol was considered and approved by the Research and Ethics Committee, Faculty of Medicine, Chiang Mai University (study number OPT-2560-04522). Patients and public involvement were not directly involved in the design of this study.

The medical records of OGI patients who were admitted from January 2006 to December 2016 were evaluated. Then, the records of injuries related to traffic accidents were identified and reviewed in detail. The collected data included patients' demographics comprising age, gender, laterality, and mechanism of injury. Ocular features noted at initial examination included visual acuity (VA), zone and location of injury, wound length, presence of relative afferent pupillary defect (RAPD), anterior and posterior segment abnormalities, presence of endophthalmitis, and associated adnexal injury. Zone of injury was classified according to ocular trauma classification into injury limited to cornea and corneoscleral limbus (Zone I), injury at anterior five mm of sclera (Zone II), and injury that extended more than five mm from the limbus into posterior sclera (Zone III) [17]. An ocular trauma score was calculated from the initial VA and presence of the following features: globe rupture, endophthalmitis, perforating injury, retinal detachment, and RAPD [18]. The mechanisms of injury were divided by the Birmingham Eye Trauma Terminology System (BETT) into 4 types: globe rupture (referred to the eye injury by blunt force), penetration and perforation (referred to eye injury by sharp force), and intraocular foreign body (IOFB) [19]. Ophthalmic management and final VA at last follow-up were evaluated. The improvement or worsening of one or more Snellen VA line at the final visit compared to the presenting visit was evaluated.

### 2.1. Statistical Analyses

Patients' demographics were presented by descriptive analysis. Categorical data comparison was performed by the chi-squared or Fischer exact test. For continuous data comparison, Kruskal–Wallis test, and Wilcoxon signed-rank test were used as appropriate. Snellen VA was converted to the Logarithm of the Minimum Angle of Resolution (LogMAR) for VA analysis. Poor final VA was defined as the Snellen VA worse than 20/400. Multivariable regression analysis for poor final VA was adjusted for age, gender, initial VA, mechanisms of injury, uveal tissue prolapse, vitreous prolapse, presence of retinal detachment, presence of endophthalmitis, presence of lens injury, and presence of RAPD with the enter method. A statistical significance was considered as *P* < 0.05. All analyses were performed using SPSS version 24 (IBM Corp., Armonk, NY, USA).

## 3. Results

**Figure 1:**
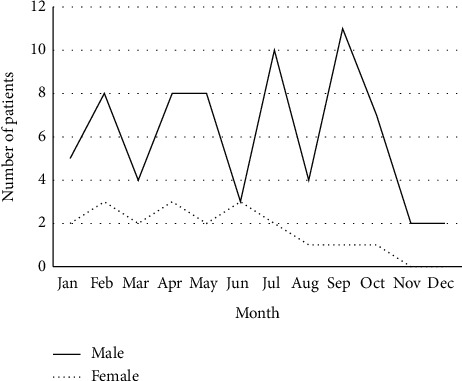
Incidence of traffic-related open globe injury by gender and month of a year.

**Figure 2:**
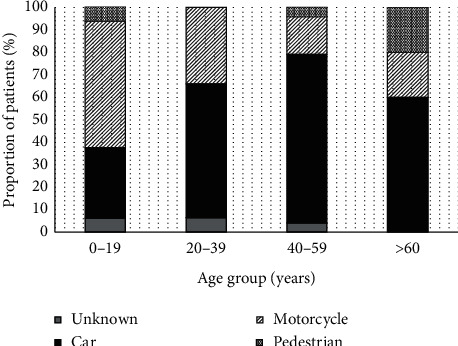
Distribution of vehicle modes involving in the injury by age groups.

From 978 hospitalized OGI cases, 92 (9.4%) patients (102 eyes) sustained injury related to a traffic accident with a median (interquartile range) follow-up of nine (3.4 to 31) months. These patients had a mean (SD) age of 33.9 (13.8) years (range, 5 to 84 years). Males represented 72/92 (78.3%) patients. The incidence of injury by month is illustrated in Figure 1 with no distinct pattern observed. Considering the frequency of injury by age groups, the peak incidence of injury (47/92, 51.1%) was noted in the 20–39-year-age group. Noticeably, a proportion of motorcycle-related accident was highest in patients aged less than 20 years (9/16, 56.3%), while automobile-related accident was predominant in other age groups, and Figure 2 Patients' demographics are documented in Table 1.

Regarding mechanisms of injury, 48/102 (47.1%) eyes had globe ruptures, followed by penetration in 46/102 (45.1%) and IOFB in 8/102 (7.8%). There were no patients with perforation. A median (interquartile range) time-lapse from injury to the hospital was 23 (5.3–114) hours. At initial examination, the median (IQR) presenting LogMAR VA was 2.3 (1.9 to 2.3) with the mean (SD) ocular trauma score of 51 (19.5). There was no statistical difference in initial VA between types of injury (*P*=0.18). Characteristics of injured eyes are presented in Table 2, and only three (3%) developed endophthalmitis.

Overall, the mean (SD) of admission duration was 10.2 (5.8) days. Primary wound repair was performed in 97/102 (95.1%) eyes; the remaining five eyes presented with self-sealed wounds. Fifty-four eyes (54/102, 52.9%) required two or more surgical interventions. Posterior segment surgery was done in 52/102 (50.9%) eyes. Enucleation/evisceration was eventually performed in 13/102 (12.7%) eyes. Secondary glaucoma was detected in 10/102 (9.8%) eyes. The median (IQR) final LogMAR VA, 1.7 (0.3 to 3.0), was significantly improved compared to the presenting VA, *P* < 0.001. The improvement of final VA was found in 56/102 (54.9%) eyes. On the contrary, 19/102 (18.6%) eyes had worsening of the final vision. Thirty eyes (29.4%) achieved final VA of 20/40 or better, whereas 27 eyes (26.5%) acquired no perception of light (NPL). In addition, among those presenting with initial NPL, 12/15 (80%) eyes remained NPL, 2/15 (13.3%) recovered to perception of light, and 1/15 (6.7%) regained to hand movement following treatments.

With multivariable regression analysis, presence of RAPD (odds ratio, 4.350; 95% confidence interval, 1.047–18.070; *P*=0.043) and presence of retinal detachment (Odds ratio, 6.862; 95% confidence interval, 1.258–37.429; *P*=0.026) were significant factors for a poor final VA as shown in Table 3.

## 4. Discussion

The impact of traffic accidents has been evaluated in several eye trauma studies in hospitalized patients. In Taiwan, Lee et al. demonstrated that the main cause for both principal (20.4%) and secondary diagnosis (47.2%) of admitted eye trauma was traffic accidents [10]. In China, Qi et al. pointed out that almost a quarter of hospitalized eye injuries (24.2%) occurred from vehicle-related accidents [8]. Nevertheless, when considering the overall incidence of traffic-related OGI with a poorer visual prognosis compared to closed globe injury, the variation in incidence according to countries has been published [7]. In high-income countries, a low incidence of traffic accidents has been shown in studies by Li et al. (2%) from Hong Kong, Beshay et al. (2%) from Australia, Fujikawa et al. (3.4%) from Japan, Court et al. (3.4%) from New Zealand, and Orr et al. (4%) from the US [2, 13, 14, 20, 21]. On the contrary, a much higher incidence has been reported in a study by Madhusudhan et al. (17.6%) from Malaysia and, accordingly, in this study (9.4%), which were both characterized as middle-income countries [12]. With a relatively high incidence of traffic-related eye injuries in this region, it is essential to have more actions from the government and community to make the road safer. However, to establish effective enforcement of a road safety system, it is reasonable to properly define the vulnerable risk groups and to investigate related consequences following eye injury.

Considering gender, the result of this study is consistent with other OGI publications, which either investigated for overall causes or only for traffic-related injury, which found that males were a major proportion of patients, even though varying in ratio [13, 15, 16, 21, 22]. This may refer to differences in physical activities between genders. Therefore, public awareness for road safety legislation should be promoted for better understanding among the population, particularly males. Apart from gender, specific age range has been observed to differ between causes of OGI. In fall-down-related OGI, the peak incidence has been described in young children and the elderly, while patients between 30 and 40 years were at increased risk for occupational-related OGI [2, 6, 15, 23]. Nevertheless, in traffic-related OGI, the disparities in age range have been demonstrated. A study in the US by Orr et al. reported that nearly half of the cases (48%) were young patients between 20 and 40 years old, whereas a study in Japan by Okamoto et al. found that traffic injury comprised a higher proportion of elderly patients with a mean age of 50 years [21, 22]. This study, similar to Zhang et al., demonstrated that the highest incidence occurred in young patients between 20 and 39 years which represented young adult and/or early working groups [9]. This finding is in line with a previous report in Thailand which showed that the most commonly admitted traffic-injured age group was 15 to 30 years old [24]. The less experienced in vehicle-related performance, and an easily distracted nature might possibly explain the high incidence of injury in this age range. Therefore, strategies to encourage the use of safety equipment and to follow the safety instructions involving vehicle transportation should be emphasized.

Types of vehicle are another factor that should be considered. A study by Orr et al. reported that nearly all cases (96%) were injured by automobiles and the minority were (4%) injured by motorcycles [21]. Okamoto et al. noted that the two most frequent injuries were related to car driving (36%) and bicycle riding (14%), while the minority were related to motorcycle riding (7%) [22]. However, this study revealed that the proportion of transportation modes varied by age range. Motorcycles were the most prevalent in the young while the automobiles were the most frequent in adults and the elderly. Regular inspection of safety behaviors during riding/driving including seat belt use, helmet use, speed limitation, and alcohol and mobile phone restrictions might benefit injury prevention across all age groups.

Regarding the mechanism of injury, there have been pieces of evidence that globe rupture was the most prevalent type (60 to 64%) in traffic-related OGI [21, 22]. This study noted that a similar proportion of patients sustained injuries from globe rupture and penetration (47 versus 45%). This may refer to the nature of traffic accidents with a higher chance for diffuse ocular damage by both blunt forces, including trauma from vehicle parts or road-related construction, and sharp penetration by objects such as glass. In contrast, most work-related OGI studies have shown that penetration was the most common mechanism [6, 23]. The high proportion of ruptures may partly explain a low visual potential. In this study, although VA was significantly improved when comparing final to initial visits, 46% of the patients' final vision was worse than 20/200. Consequently, aiming for prevention combined with prompt treatment should be an effective way to reduce severe visual impairment from traffic-related OGI.

This study had some limitations due to its retrospective design. As such, detailed information regarding patient and injury severity may have been under or overestimated. Furthermore, as this is a hospital-based study, it might not represent the population with minor injuries. However, the results point out several aspects of traffic-related OGI characteristics that should be of concern in a middle-income country.

In summary, this study provides information regarding traffic-related OGI that had a high prevalence in the young. The risky transportation modes were motorcycles in teenagers and automobiles in young adult groups. A considerable proportion of impaired final VA might have a significant impact on the socioeconomic system. Establishing effective safety education and encouraging regular adherence to road safety behaviors are challenging issues that need more action.

## Figures and Tables

**Table 1:** Demographics of road traffic-related open globe injury patients.

Characteristics of patients	Number, *N* (%)
Gender	
Male	72 (78.3)
Female	20 (21.7)

Age group	
<20 years	16 (17.4)
20–39 years	47 (51.1)
40–59 years	24 (26.1)
≥60 years	5 (5.4)

Laterality	
Unilateral	82 (89.1)
Bilateral	10 (10.9)

Status	
Pedestrian	3 (3.3)
Car driver/passenger	54 (58.7)
Motorcycle rider/passenger	30 (32.6)
Unknown	5 (5.4)

Causative objects (102 eyes)	
Broken glass	42 (41.2)
Blunt object/vehicle part	39 (38.2)
Metal object	8 (7.8)
Wood	2 (2.0)
Unknown	11 (10.8)

**Table 2:** Ocular characteristics of road traffic-related open globe injury.

Characteristics	Eye, *N* (%)
Zone of injury	
I	21 (20.8)
II	41 (40.6)
III	39 (38.6)

Wound length	
**≤**10 mm	50 (49.5)
**>**10 mm	49 (48.5)
Unknown	2 (2.0)

Initial VA	
20/40 and better	6 (5.9)
20/50 to 20/200	4 (3.9)
19/200 to projection of light	73 (71.5)
No perception of light	16 (15.7)
Cannot be assessed	3 (2.9)

Presence of RAPD	45 (50.6)
Hyphema	56 (55.4)
Lens injury	53 (52.5)
Uveal tissue prolapse	56 (55.4)
Vitreous prolapse	31 (30.7)
Vitreous hemorrhage	51 (50.5)

Retinal injury	
Retinal detachment	36 (35.6)
Retinal break	6 (5.9)
Commotio retinae	2 (2.0)
Choroidal injury	22 (21.8)
Adnexal injury	
Eye lid injury	37 (36.6)
Orbital injury	7 (6.9)
Both lid and orbital injury	13 (12.9)
Presence of endophthalmitis	3 (3.0)

Ocular trauma score class	
Class 1 (0–44)	29 (28.7)
Class 2 (45–65)	41 (40.6)
Class 3 (66–80)	23 (22.8)
Class 4 (81–91)	2 (2.0)
Class 5 (92–100)	3 (3.0)
Cannot be assessed	3 (3.0)

VA = visual acuity; RAPD = relative afferent pupillary defect.

**Table 3:** Multivariable analysis for the factors related to poor visual outcome.

	Odds ratio	95% confident interval	*P* value

Age	1.023	0.972 to 1.076	0.389
Sex	0.517	0.119 to 2.254	0.380
Initial VA ≤20/400	6.928	0.784 to 61.230	0.082
Mechanisms of injury			
IOFB	—	—	—
Rupture	0.994	0.100 to 9.911	0.996
Penetrating	0.156	0.016 to 1.545	0.112
Lens injury	0.674	0.174 to 2.609	0.568
Uveal tissue prolapse	1.148	0.399 to 5.516	0.557
Vitreous prolapse	0.371	0.105 to 1.315	0.125
Retinal detachment	4.350	1.258 to 37.429	0.026
Presence of RAPD	3.857	1.047 to 18.070	0.043

IOFB = intraocular foreign body; RAPD = relative afferent pupillary defect.

## Data Availability

The data used to support the findings of this study are available from the corresponding author upon request.

## Abbreviations

BETT: The Birmingham Eye Trauma Terminology System
IOFB: Intraocular foreign body
IQR: Interquartile range
LogMAR: Logarithm of the minimum angle of resolution
NPL: No perception of light
OGI: Open globe injury
RAPD: Relative afferent pupillary defect
SD: Standard deviation
VA: Visual acuity.
